# Assessing the prospect of XAFS experiments of metalloproteins under *in vivo* conditions at Indus-2 synchrotron facility, India

**DOI:** 10.1107/S1600577522011791

**Published:** 2023-01-13

**Authors:** Debdutta Lahiri, Richa Agrawal, Khileshwari Chandravanshi, Parasmani Rajput, Ankur Agrawal, Ashutosh Dwivedi, Ravindra D. Makde, S. N. Jha, Nandini Garg

**Affiliations:** a High Pressure and Synchrotron Radiation Physics, Bhabha Atomic Research Centre, Mumbai 400085, India; b Department of Biochemistry and Molecular Biology, University of Chicago, 929 E 57th Street, Chicago, IL 60637, USA; c Beamline Development and Application Section, Bhabha Atomic Research Centre, Mumbai 400085, India; d Homi Bhabha National Institute, Training School Complex, Anushaktinagar, Mumbai 400094, India; University of Essex, United Kingdom

**Keywords:** XAFS, metalloproteins

## Abstract

The feasibility of X-ray absorption fine-structure (XAFS) experiments of ultra-dilute metalloproteins under *in vivo* conditions (*T* = 300 K, pH = 7) at the BL-9 bending-magnet beamline (Indus-2) is reported, using as an example analogous synthetic Zn (0.1 m*M*) M1dr solution. The (Zn *K*-edge) XAFS of M1dr solution was measured with a four-element silicon drift detector. The first-shell fit was tested and found to be robust against statistical noise, generating reliable nearest-neighbor bond results. The results are found to be invariant between physiological and non-physiological conditions, which confirms the robust coordination chemistry of Zn with important biological implications. The scope of improving spectral quality for accommodation of higher-shell analysis is addressed.

## Abstract 2

The feasibility of X-ray absorption fine-structure (XAFS) experiments of ultra-dilute metalloprotein solutions at Indus-2 is evaluated.

## 1. Introduction

Metalloproteins (MPs) represent one of the most diverse classes of proteins, with the intrinsic metal atoms providing catalytic, regulatory or structural roles critical to protein function (https://www.sciencedirect.com/topics/chemistry/metalloprotein). They are at the heart of diverse biological processes related to disease propagation, *e.g.* gene regulation, protein matrix degradation, antibiotic resistance. Therefore, research of MPs understandably occupies center stage in the contemporary battle against diseases, with the aim of understanding the origin of the diseases, the functioning of drugs, drug resistance and discovery of new drugs (Cho *et al.*, 2017 ▸). The intricate nature of biochemical reactions in living cells demands high specificity, which is defined by the geometrical and chemical precision of the metal-binding with amino acid residues of the protein. Thus, determination of the coordination chemistry of the metal is critical to the understanding of MP functioning. The structural aspect of this problem is solved (within 1.2 Å resolution) for MP crystals at low temperature (*i.e.* under non-physiological conditions) by employing synchrotron-based X-ray diffraction crystallography (XRD) (Yamamoto *et al.*, 2017 ▸; Petrova & Podjarny, 2004 ▸; Shi, 2014 ▸). However, the scope of crystallography is fundamentally limited due to its insensitivity to chemical state and amorphous structure. This precludes chemical speciation of metals and structural determination of MPs under real *in vivo* conditions (*e.g.* solution form at room temperature). Besides, practical problems with XRD emanate from (i) the reliance upon high-quality crystals that are difficult to fabricate and (ii) the inability to monitor the real-time chemical state of the metal that could be susceptible to synchrotron radiation (Weik *et al.*, 2000 ▸; Corbett *et al.*, 2007 ▸; O’Neill *et al.*, 2002 ▸). These limitations of XRD necessitate alternative techniques with sensitivity to amorphous structure and chemical state, both of which are met by X-ray absorption fine structure (XAFS) (Koningsberger & Prins, 1988 ▸).

X-ray absorption spectroscopy measures the absorption of X-rays in materials as a function of incident X-ray energy (Koningsberger & Prins, 1988 ▸). The atom of interest is excited by tuning the X-ray energy to its binding edge (*E*_0_), which makes this technique element-specific. XAFS is based on interference between the ejected photoelectron and its backscattered counterpart (by neighboring atoms). Since the coherence of the electron waves underlines the interference phenomenon, XAFS information is localized within the coherence length of the electron (λ ≃ 10 Å). This essentially eliminates long-range-order dependence and means the technique is sensitive to amorphous structure. Near-neighbor species [*Z* (±5)], coordination number [*N* (±1)], radial distance [*R* (±0.01 Å)] and disorder [σ^2^ (±0.001 Å^2^)] information are retrieved from XAFS analysis (Lahiri, 2008 ▸). X-ray absorption near-edge structure (XANES) is the portion of the XAFS spectrum near an absorption edge, that is sensitive to the chemical state and coordination geometry through modulations of (i) the edge energy (*E*_0_) increases with higher oxidation state (Pantelouris *et al.*, 1995 ▸), (ii) the intensity of the first post-edge peak or ‘white-line’ (Brown *et al.*, 1977 ▸) – proportional to the oxidation state, and (iii) the pre-edge peak intensity – sensitive to the coordination-symmetry controlled *pd* orbital hybridization (Shishido *et al.*, 2009 ▸). Thus, XAFS is collectively capable of reconstructing the metal–ligand coordination chemistry of MPs (also under *in vivo* conditions) that has inspired its integration into the MPs problem (Ascone *et al.*, 2005 ▸; Shi *et al.*, 2011 ▸; Cotelesage *et al.*, 2012*a* ▸,*b* ▸; Strange *et al.*, 2005 ▸).

India is home to endemic diseases (*e.g.* malaria, tuberculosis, hepatitis), which involve MPs (Goldberg *et al.*, 1990 ▸; Gonçalves *et al.*, 2017 ▸; Chim *et al.*, 2014 ▸; Tellinghuisen *et al.*, 2004 ▸). Research of the relevant MPs is therefore a prime scientific mandate of the Government of India. A protein crystallography beamline (Kumar *et al.*, 2016 ▸) has been commissioned at the Indus-2 (2.5 GeV) synchrotron facility in India (https://www.rrcat.gov.in/technology/accel/indus2.html) to this effect. Recognizing the parallel importance of XAFS for MPs, it was proposed to be initiated at the existing bending-magnet-based XAFS beamline BL-9 (https://www.rrcat.gov.in/technology/accel/srul/beamlines/exafsscan.html). Bio-XAFS experiments are amongst the most challenging (Ortega *et al.*, 2012 ▸), due to the inherent limitations of low metal concentration (m*M*) (Ranieri-Raggi *et al.*, 2003 ▸), large disorder and susceptibility to radiation damage (Weik *et al.*, 2000 ▸; Corbett *et al.*, 2007 ▸; O’Neill *et al.*, 2002 ▸). This mandates advanced supportive technologies, *e.g.* high photon flux (Fischetti *et al.*, 2004 ▸; Gauthier *et al.*, 1999 ▸; Cotelesage *et al.*, 2012*a* ▸,*b* ▸; Proux *et al.*, 2005 ▸; Adachi *et al.*, 2001 ▸) and efficient fluorescence detectors (Cramer *et al.*, 1988 ▸) for high signal statistics, fast scanning monochromators for short exposure time (Khalid *et al.*, 2011 ▸) and cryo-cooling for arresting radiation damage (Ramanan *et al.*, 2015 ▸). These experimental facilities are moderately satisfied at BL-9, *e.g.* flux ≃ 10^11^ photons s^−1^, four-element silicon VORTEX detector (Barkan *et al.*, 2003 ▸), QEXAFS (Poswal *et al.*, 2016 ▸) and cryo-cooling (Ramanan *et al.*, 2015 ▸), which encouraged XAFS measurements of Cu protein (powder) at this beamline (Dutta Gupta *et al.*, 2021 ▸).

**Figure 1:**
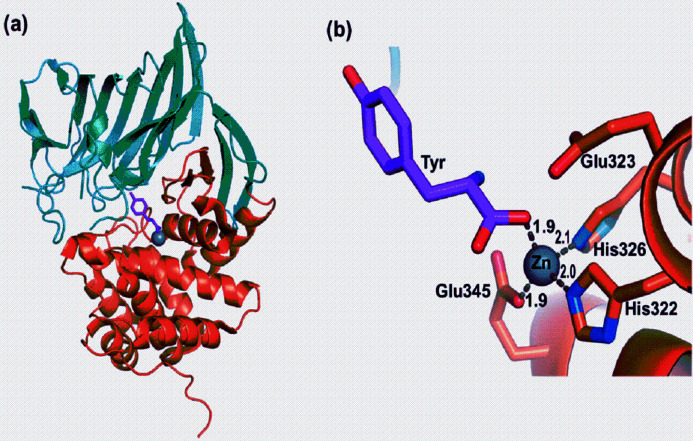
(*a*) Crystal structure of M1dr at 77 K. (*b*) Zn-binding sub-unit of M1dr.

Following the first successful experiments, we undertook a realistic assessment of the scope of XAFS of MPs under *in vivo* conditions at BL-9, *e.g.* in solution form, down to ultra-dilute concentrations. This task includes actual XAFS measurement of ultra-dilute MP solution at BL-9 and evaluation of spectral quality, reliability of results, the scope of advanced analysis and technological suggestions for improvement. In this paper, we present such evaluation with the example of Zn *K*-edge XAFS of analogous synthetic Zn (0.1 m*M*) M1dr solution at room temperature (Agrawal *et al.*, 2019 ▸). M1 is a protease from *Deinococcus radiodurans* (Uniprot ID: Q9RVZ5) – radioresistant bacterium. M1dr belongs to the M1 Zn metallo­protease family whose sequence homologs are involved in tumor growth, angiogenesis, hormone regulation, immune cell development and Huntington’s disease (Kelly *et al.*, 1997 ▸). Structural pre-determination for the crystal counterpart of M1dr (with XRD) (Agrawal *et al.*, 2019 ▸) provided the reference for the XAFS reliability test [Figs. 1 ▸(*a*), 1 ▸(*b*)]. XRD of an M1dr crystal (*T* = 77 K) revealed a tetrahedral configuration of Zn, forming bonds with (N, O) bridging atoms of His322, His326 and Glu345 [Fig. 1 ▸(*b*)]. The choice of Zn protein (vis-à-vis other metals) was inspired by a few factors: (i) its ubiquitous biological importance, since Zn is the second most abundant transition metal in organisms and the only metal present in all enzyme classes (Kreźel & Maret, 2016 ▸; Maret, 2013 ▸) – therefore, XAFS evaluation for any *one* Zn MP (*e.g.* M1dr) would potentially represent a wide range of MP systems based on Zn; (ii) XAFS assumes particular importance as the only probe for Zn-proteins, since Zn^2+^ is inaccessible to other spectroscopic techniques due to its filled 3*d* level (Penner-Hahn, 2005 ▸); (iii) from a practical perspective, Zn-protein serves as a good benchmark for XAFS feasibility tests because of prior extensive XAFS investigations and structural cataloging (Bobyr *et al.*, 2012 ▸; Dent *et al.*, 1990 ▸; Feiters *et al.*, 2003 ▸; Murphy *et al.*, 1997 ▸; Tierney & Schenk, 2014 ▸; Kleifield *et al.*, 2001 ▸; Amiss & Gurman, 1999 ▸; Meyer-Klaucke *et al.*, 1999*a* ▸; Giachini *et al.*, 2007 ▸, 2010 ▸; Clark-Baldwin *et al.*, 1998 ▸; Christianson, 1991 ▸; Pace & Weerapana, 2014 ▸; Laitaoja *et al.*, 2013 ▸); (iv) *K*-edge XANES is a good marker for XANES calibration due to the fixed and stable (against radiation) Zn^2+^ state (Giachini *et al.*, 2010 ▸; Penner-Hahn, 2005 ▸; Al-Ebraheem *et al.*, 2010 ▸; Castorina *et al.*, 2019 ▸).

Our experiments were statistically challenged by one-order-of-magnitude lower metal concentration and the unavailability of a standard multi-element germanium detector (Bobyr *et al.*, 2012 ▸; Dent *et al.*, 1990 ▸; Feiters *et al.*, 2003 ▸; Murphy *et al.*, 1997 ▸; Tierney & Schenk, 2014 ▸; Kleifield *et al.*, 2001 ▸; Amiss & Gurman, 1999 ▸; Meyer-Klaucke *et al.*, 1999*a* ▸,*b* ▸; Giachini *et al.*, 2007 ▸, 2010 ▸; Clark-Baldwin *et al.*, 1998 ▸; Christianson, 1991 ▸; Pace & Weerapana, 2014 ▸; Laitaoja *et al.*, 2013 ▸). Nonetheless, we undertook this challenge with the understanding that a feasibility test under the worst experimental conditions warrants foolproof credibility. We met the challenge with strategies such as (*a*) a large sample area by injecting solution inside an (X-ray transparent) Kapton bag (https://www.dupont.com/electronic-materials/kapton-polyimide-film.html); (*b*) XAFS measurement in fluorescence mode with a four-element silicon VORTEX detector (equipped with fast electronics) (Barkan *et al.*, 2003 ▸); (*c*) shielding of the detector from stray photons; (*d*) minimization of the sample–detector distance, and (*e*) iterative data collection on fresh solutions, to guard against radiation damage. No radiation damage was shown between successive scans, as the Zn^2+^ state remained stable between XANES scans. Our strategies generated reproducible Zn *K*-edge XAFS spectra up to Δ*E* = +400 eV past the edge (*k* = 11 Å^−1^). Although spectral range and quality are statistically compromised (as speculated), the Fourier transform of the XAFS spectra over *k* = 2.5–10 Å^−1^ generated a reproducible first-shell peak over *R* = 0.8–2 Å and permitted reliable first-shell analysis. XAFS analysis reproduced coordination and bond-length (and distribution) results of XRD (Agrawal *et al.*, 2019 ▸), within intrinsic analytic uncertainty. Negligible evolution of the coordination chemistry of M1dr between low-temperature crystal (*T* = 77 K, pH 5.5) (Agrawal *et al.*, 2019 ▸) and *in vivo* (*T* = 300 K, pH 7) conditions demonstrates robustness of Zn—(O/N) bonds. This robustness resembles the behavior of three- and four-domain proteins of the M1 family and accounts for efficient substrate binding in the absence of the C-domain (Agrawal *et al.*, 2019 ▸). Thus, novel perspectives of M1dr are unraveled by this experiment.

Our success confirms the feasibility of XAFS of MPs solution at Indus-2 BL-9, down to ultra-dilute concentrations. Since the beamline is capable of delivering X-rays in the energy range 5–20 keV, a plethora of metals (*Z* = 23–42; ≥53) can be probed, covering a wide range of MPs (and organometallics in general). XANES and first-shell results can be reliably obtained for these MPs that would provide information on the chemical state of the metal, identity of ligand groups, (N/O):S coordination ratio and geometric distortion (Bobyr *et al.*, 2012 ▸; Dent *et al.*, 1990 ▸; Feiters *et al.*, 2003 ▸; Amiss & Gurman, 1999 ▸; Meyer-Klaucke *et al.*, 1999*a* ▸,*b* ▸; Giachini *et al.*, 2010 ▸; Clark-Baldwin *et al.*, 1998 ▸; Christianson, 1991 ▸; Pace & Weerapana, 2014 ▸; Laitaoja *et al.*, 2013 ▸; Smolentsev *et al.*, 2005 ▸; Longa *et al.*, 1999 ▸; Vlasenko *et al.*, 1999 ▸; Sagi *et al.*, 1999 ▸; Katsikini *et al.*, 2009 ▸; Bertoncini *et al.*, 1999 ▸). This information can adequately address diverse biological problems such as disease-marking, binding properties, protein aggregation, multi-site heterogeneity, mutation and cellular catalysis (Smolentsev *et al.*, 2005 ▸; Longa *et al.*, 1999 ▸; Vlasenko *et al.*, 1999 ▸; Sagi *et al.*, 1999 ▸; Katsikini *et al.*, 2009 ▸; Meyer-Klaucke *et al.*, 1999*a* ▸; Bertoncini *et al.*, 1999 ▸). The prospect of improving the spectral quality of XAFS, to accommodate higher-shell-based novel scientific problems (Giachini *et al.*, 2007 ▸; Kleifield *et al.*, 2001 ▸; Murphy *et al.*, 1997 ▸; Tierney & Schenk, 2014 ▸), is addressed. This work should inspire *in vivo* XAFS experiments of MPs at beamlines with modest facilities like ours.

## 2. Experimental details

### 2.1. Sample preparation

**Figure 2:**
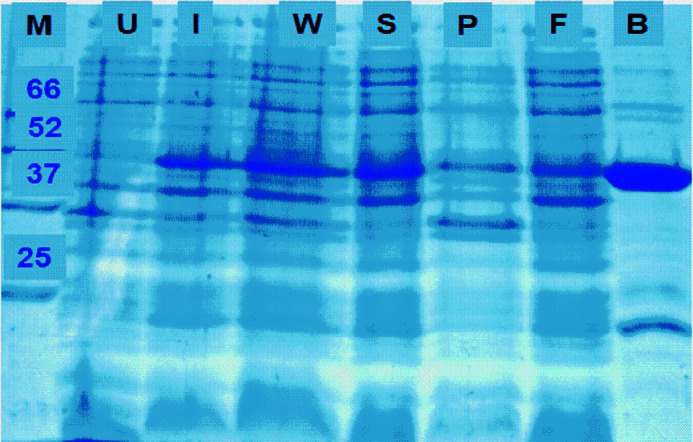
SDS gel image of M1dr Zn-NTA purification. M = protein marker (numbers shown in kDa), (U)I = (Un)introduced, W = cell lysate, S = supernatant after spin, P = pellet after spin, F = flow through after Zn binding, B = bound M1 protein.

M1dr protein was expressed in Rosetta(DE3)pLysS *E.coli* expression host and purified from cell lysate through Ni-NTA chromatography using 50 m*M* phosphate buffer pH 7 (Agrawal *et al.*, 2019 ▸). Purified protein was stored at −80°C with 20% glycerol v/v (Fig. 2 ▸). This was mixed with 0.1 m*M* ZnCl_2_ externally and spun at 12000 rpm for 10 min, prior to XAFS experiment. The solution was injected into a large Kapton bag and sealed for XAFS measurement.

### 2.2. Experimental setup for XAFS

**Figure 3:**
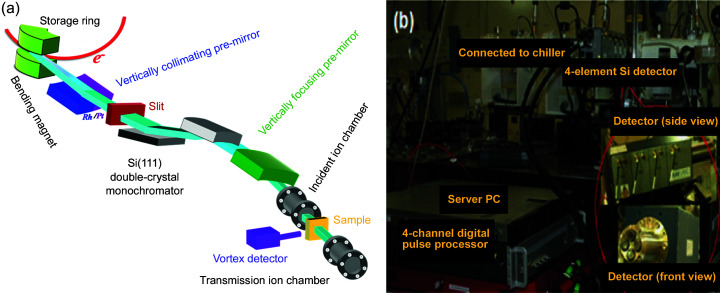
(*a*) Schematic outline (adapted from the RRCAT website) and (*b*) photograph of the XAFS beamline BL-9 at Indus-2.

A schematic layout and photograph of BL-9 are depicted in Figs. 3 ▸(*a*) and 3 ▸(*b*). The beamline is designed to deliver monochromatic X-rays of energy ∼5–20 keV and flux ∼10^11^ photons s^−1^ at the sample position. A Si(111) double-crystal monochromator, consisting of a water-cooled first crystal and horizontally focusing second crystal, was aligned for monochromatic X-rays around the Zn *K*-edge (9.659 keV). Higher harmonics were rejected and the beam vertically collimated by a Rh-coated meridional cylindrical pre-mirror. The final spot size at the sample position was approximately 1 mm (H) × 0.2 mm (V). For reference, XAFS for Zn foil and ZnO powder were measured in transmission with gas-filled ion chambers. Mixtures of helium/nitrogen and nitrogen/argon gases were respectively filled in incident and transmission ion chambers. XAFS for Zn foil was used for energy calibration of the monochromator.

XAFS of M1dr solution (inside the Kapton bag) was measured in fluorescence mode, due to the dilute metal content (https://xafs.xrayabsorption.org/tutorials.html). A gas-filled ion chamber and silicon drift detector (SDD) were employed for monitoring the intensities of the incident and (Zn 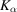 ) fluorescence photons, respectively. The SDD was mounted on a (remote-controlled) motorized *xyz* stage and adequately shielded against stray photons.

### 2.3. Fluorescence detector

The choice of SDD played a key role in the improvement of the XAFS data quality. A single-element SDD (active area = 50 mm × 50 mm, collimated area = 30 mm × 30 mm) was initially employed but its statistical inefficiency due to low input count-rate (ICR ≃ 10^6^) and high dead-time generated poor signal. This problem was overcome with the installation of an efficiently designed four-element SDD, that was geometrically and electronically adapted for high-quality signal (https://www.rayspec.co.uk/content/uploads/2016/12/4-Page-RaySpec-Beamlines.pdf). Four sensors of the SDD (each active area = 40 mm × 40 mm, collimated area = 30 mm × 30 mm) are located on the surface of a (virtual) sphere, centered at the sample. This geometry generates equal solid angles for the four sensors, so that they are uniformly illuminated and the total solid angle of the detector is increased fourfold. This leads to a fourfold increase of the fluorescence photon collection efficiency. A digital pulse processor of the single-element SDD was replaced by a four-channel Xspress-3 readout with high ICR (= 3.5 × 10^6^ counts s^−1^) and 20% dead-time (https://quantumdetectors.com/products/xspress3/), which enabled handling of 12–14 times higher photon flux. These upgrades jointly promoted the efficient utilization of beam flux. The readout has been integrated with a data acquisition system and GUI developed to automatically configure detector parameters (*e.g.* acquisition time, calibration factor) through an EPICS–LabVIEW interface.

### 2.4. Data collection

XAFS spectra were acquired in steps of (i) 10 eV (1 s) over the pre-edge, (ii) 0.5 eV (1 s) over the XANES and (iii) 0.05 Å^−1^ (15 s) over the EXAFS regions (Kane *et al.*, 2014 ▸). Iterations were limited to (×3) scans due to time constraints. [Several diagnostic tests were exercised to pre-determine the optimal experimental setup. These included evaluation of data quality for various concentrations of Zn samples, sample holders (Kapton bag vis-à-vis cuvet) and detectors (single vis-à-vis four-element SSD).]

## 3. Results and discussions

### 3.1. XANES

**Figure 4:**
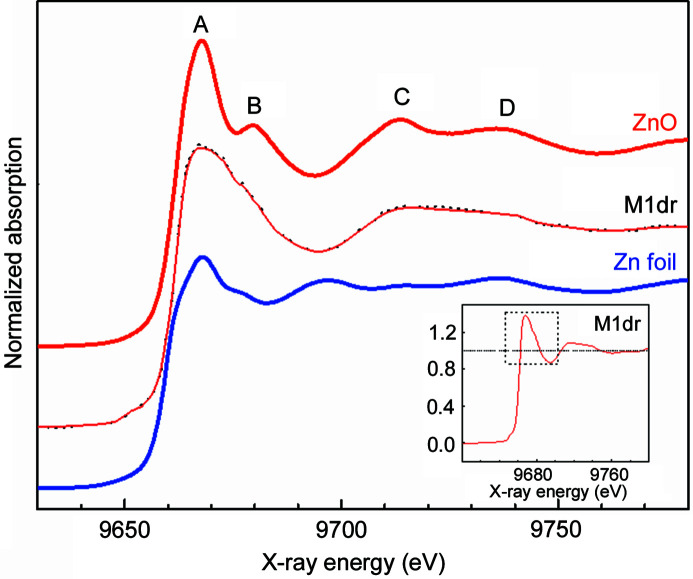
Normalized Zn *K*-edge XANES spectra for Zn foil and ZnO powder standards and for M1dr solution. Datasets are shifted relative to each other for clarity. Raw (black dotted) and smoothened (pink solid) datasets for M1dr are overplotted. Inset: magnified image of the white line for M1dr. White-line intensity = 1.35 suggests *N* = 4 coordination of Zn.

Zn *K*-edge XAFS data μ(*E*) were processed using *ATHENA* software (Ravel & Newville, 2005 ▸). Datasets for M1dr solution were reproducible, within statistical fluctuations. The signal-to-noise ratio could be ideally improved with 10–15 scans. However, the number of iterations was limited to ×3 in our case, due to time constraints. The average of the ×3 datasets was smoothened by the interpolative smoothing algorithm of *ATHENA* with three iterations. Fig. 4 ▸ displays normalized Zn *K*-edge XANES spectra for standards [Zn foil (Zn^0^), ZnO powder (Zn^2+^)] and M1dr solution. An overplot of the smoothed and original dataset for M1dr (Fig. 4 ▸) rules out the scope of data distortion, as far as XANES and first-shell EXAFS analysis are concerned. Henceforth, the smoothened dataset was used for analysis. XANES data were analyzed for (i) edge energy (*E*_0_) and (ii) white-line intensity.

(i) The edge energy (*E*_0_) was defined at the half-point of the rising edge of the absorption curve. XANES spectra of the standards demonstrate a positive shift of *E*_0_: 9659 eV (Zn) → 9659.9 eV (ZnO), consistent with increasing oxidation state. For M1dr, *E*_0_ = 9659.9 eV coincides with *E*_0_ for ZnO. [In principle, *E*_0_ can also be defined at the point of inflection of XANES derivative spectra. In this work, normalized XANES spectra (rather than derivative) are presented to enable calibration of Zn ligand coordination with white-line intensity (Penner-Hahn, 2005 ▸; Al-Ebraheem *et al.*, 2010 ▸; Castorina *et al.*, 2019 ▸).]

(ii) White-line (WL) features (*A*, *B*) represent the probability of 1*s* → 4*p* electronic transitions (Koningsberger & Prins, 1988 ▸). The WL is significantly pronounced from Zn to ZnO, consistent with the increased availability of empty *p* states due to lower electron content. XANES features beyond *A*, *B* are distinct between Zn and ZnO. XANES peaks (*A*, *B*, *C*, *D*) for M1dr resemble the peaks of ZnO with respect to the centroid and relative intensity (except for overall broadening due to disorder).

Thus, both *E*_0_ and WL jointly confirm the Zn^2+^ oxidation state for M1dr.

Besides the oxidation state, the WLs for Zn MPs are reportedly sensitive to ligand coordination (*N*) *via* the density of states (Penner-Hahn, 2005 ▸; Al-Ebraheem *et al.*, 2010 ▸; Castorina *et al.*, 2019 ▸). Standardized correlation between WL and *N* sets the criterion: WL < 1.5 ⇒ *N* = 4. By this criterion, WL = 1.35 for M1dr (magnified in the inset of Fig. 4 ▸) unambiguously confirms *N* = 4, consistent with the XRD model (Agrawal *et al.*, 2019 ▸). We remark that the conventional pre-edge peak for tetrahedral geometry (corresponding to the *s* → *d* transition) is absent in Zn *K*-edge XANES, since the *s* → *d* transition is forbidden for Zn^2+^ due to the fully occupied *d*-shell of Zn^2+^.

### 3.2. EXAFS

**Figure 5:**
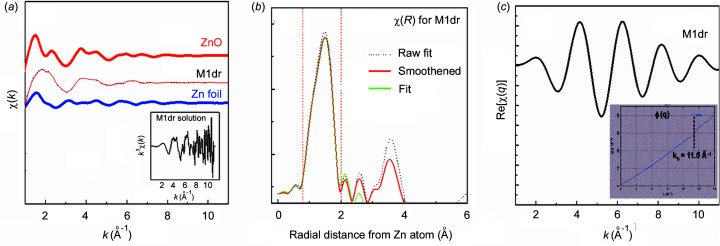
(*a*) Zn *K*-edge XAFS oscillations χ(*k*) for Zn foil and ZnO powder standards and for M1dr solution at room temperature, laterally shifted relative to each other for clarity. Raw (black dotted) and smoothened (pink solid) datasets for M1dr are overplotted. Inset: *k* ^3^χ(*k*) for M1dr highlights noise over the higher *k*-region. (*b*) Fourier transform of |χ(*R*)| of XAFS data for M1dr, over transform range *k* = 2.5–10 Å^−1^. |χ(*R*)| for raw (black dotted) and smoothened (pink solid) data are overplotted. The fit |χ(*R*)| (solid green) is compared. The fit range *R* = 0.8–2 Å is marked by red vertical lines. (*c*) χ(*q*) = back-transform of χ(*R*) over the first shell (*R* = 0.8–2 Å). A beat-like feature of χ(*q*) is evident. Inset: φ(*q*) = phase of χ(*q*), displaying a jump at *k*_b_ = 11.5 Å^−1^.

Normalized XAFS data of Fig. 4 ▸ were background-subtracted to derive the XAFS oscillations χ(*k*) shown in Fig. 5 ▸(*a*). χ(*k*) for M1dr solution decays fast, consistent with the presence of large disorder and the absence of high-*Z* backscattering neighbors. Raw *k* ^3^χ(*k*) for M1dr are presented in the inset of Fig. 5 ▸(*a*), for comparison of the spectral quality with reported data of Zn proteins (Meyer-Klaucke *et al.*, 1999*a* ▸; Dent *et al.*, 1990 ▸; Giachini *et al.*, 2007 ▸, 2010 ▸; Murphy *et al.*, 1997 ▸; Shi *et al.*, 2011 ▸; Bobyr *et al.*, 2012 ▸; Feiters *et al.*, 2003 ▸; Clark-Baldwin *et al.*, 1998 ▸; Amiss & Gurman, 1999 ▸). Raw *k* ^3^χ(*k*) for M1dr are dominated by noise beyond *k* = 8 Å^−1^ whereas the reported spectra retain good quality up to *k* ≃ 11 Å^−1^. This disparity may be attributed to the relative efficiencies of the four-element SDD (vis-à-vis the multi-element germanium detectors employed in the reported experiments). Fourier transforms |χ(*R*)| of raw and smoothed data for M1dr (over the transformation range *k* = 2.5–10 ^−1^) are presented in Fig. 5 ▸(*b*). They are similar over *R* = 0.8–2 Å, confirming that the first shell is negligibly contaminated by noise. This is consistent with the fact that XAFS oscillations of low-*Z* neighbors decay fast with increasing *k*. Thus, the first shell for M1dr may be concluded to be reasonably robust against noise. In contrast, higher-shell features of raw and smoothed |χ(*R*)| are rendered irreproducible by noise. It is impractical to attempt quantitative fitting of the higher shell for such a (noisy) dataset. Therefore, analysis was henceforth focused on the first-shell fit of (smoothed) χ(*k*), using the *FEFFIT* program (Ravel & Newville, 2005 ▸).

The reference first-shell structure for M1dr in Fig. 1 ▸(*b*) is derived from XRD (Agrawal *et al.*, 2019 ▸): Zn–O (×2), *R* = 1.9 Å; Zn–N (×1), *R* = 2.0 Å; Zn–N (×1), *R* = 2.1 Å. The reliability of the XAFS fit results will be ultimately tested against this distribution. XAFS fitting of this distribution presents two complications, described in the following paragraphs.

(i) *Intrinsic deviation of XAFS results from geometric distribution*. The geometric equivalent of the above distribution is *N* = 4 with mean bond-length *R* = 1.975 Å and bond-length distribution σ^2^ = 0.009 Å^2^. In principle, XAFS is expected to reproduce these values. However, these values may not be reproduced in reality, since XAFS is essentially an interference phenomenon. Scattering contributions (χ_*i*_) for closely spaced bond-lengths (as in the above distribution) can be slightly out of phase and partially cancel each other, so that the net spectra χ_tot_ 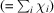 is of lower amplitude and phase-shifted. This represents lower effective coordination and/or shifted mean bond-length (Lahiri *et al.*, 2014 ▸), relative to the geometric distribution. This defines the intrinsic uncertainty of XAFS results, which has to be taken into account for meaningful comparison of XAFS and XRD results.

We theoretically pre-estimated this mismatch for the atomic distribution of M1dr *via* (*a*) simulation of χ_*i*_ for the crystallographic distribution, by exercising the ‘NOFIT’ handle of the *FEFFIT* program; (*b*) generation of a synthetic dataset χ_tot_ 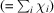 and (*c*) fitting of χ_tot_ with *N*_ZnO_, *R*_ZnO_ and 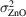 variables (assuming that O, N have similar backscattering factors). Fit results (*R*_ZnO_ = 1.94 Å, 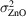 = 0.007 Å^2^) deviated slightly from the geometric equivalent (*R*_ZnO_ = 1.975 Å, 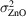 = 0.009 Å^2^). The deviations (|Δ*R*| = 0.035 Å, |Δσ^2^| = 0.002 Å^2^) thus define the intrinsic uncertainty of XAFS results for M1dr.

(ii) *Degeneracy of models, *e.g.* single-neighbor type (O/N) vis-à-vis both neighbor types (O + N)*. This problem arises due to similar backscattering factors of O and N. In principle, the degeneracy could be resolved by exploiting large bond-length differences (*e.g.* Zn—O < Zn—N). For small bond-length differences (like for M1dr), first-shell fitting (by itself) is unable to resolve the degeneracy (Giachini *et al.*, 2007 ▸; Dent *et al.*, 1990 ▸; Clark-Baldwin *et al.*, 1998 ▸). {The degeneracy can be reduced with higher-shell XAFS analysis. For example, Zn—O and Zn—N bonds could form distinct angles with second-shell atoms: Zn—O—O and Zn—N—O. Such angular disparity can be exploited to resolve the degeneracy, *e.g.* through determination of angles with multiple-scattering-based XAFS fitting (Haskel, 1998 ▸). In our case, the scope of such analysis is precluded by the domination of noise at higher *k* [see inset of Fig. 5 ▸(*a*)].}

We proceeded with first-shell XAFS analysis of M1dr with the following understandings. The fitting was designed for a single ZnO path of coordination *N*, mean bond-length *R* and spread σ^2^. XAFS for the first shell of M1dr [χ(*q*), Fig. 5 ▸(*c*)] was filtered out from the whole spectrum of Fig. 5 ▸(*a*) by back-transforming χ(*R*) over *R* = 0.8–2 Å. The presence of beats in χ(*q*) indeed confirms the presence of closely spaced multiple bond-lengths, consistent with the crystallographic model of M1dr. A phase derivative method (Piamonteze *et al.*, 2005 ▸) was employed to obtain an independent estimate of the bond-length split (Δ) from the phase φ(*q*) of the XAFS [inset of Fig. 5 ▸(*c*)]. The inflection position *k*_b_ (∼11.5 Å^−1^) of φ(*q*) is related to Δ (= π/2*k*_b_); *k*_b_ ≃ 11.5 Å^−1^ ⇒ Δ ≃ 0.13 Å, which is close to the crystallographic standard deviation of bond lengths. We remark that, as the transform range of χ(*k*) (*k* = 2.5–10 Å^−1^) bypasses *k*_b_, the spatial resolution is reduced to the extent that split ZnO peaks become indistinguishable in χ(*R*) of Fig. 5 ▸(*b*). This warranted first-shell fitting with a *single* ZnO path.

A (smoothened) XAFS dataset for M1dr was fit over *k* = 2.5–10 Å^−1^, *R* = 0.8–2 Å. The strategy of a simultaneous fit for *k* ^*w*=0–2^-weighted transforms was adopted, in order to decouple correlations between variables [(*N*, σ^2^), (*R*, Δ*E*_0_)] and minimize uncertainties in fit results [Δ*E*_0_ = energy correction, relative to edge position (*E*_0_)]. The contribution of the background was corrected by exercising the ‘bkg’ option of the *FEFFIT* program. 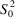 = 0.87 was pre-determined by fitting XAFS for reference Zn foil with the constraint *N*_ZnZn_ = 12 (Kelly *et al.*, 2009 ▸). Preliminary (*N*, *R*, σ^2^) fit results were refined by constraining *N* = 4 (consistent with XRD), leading to the best-fit results: *R* = 1.953 (1) Å, σ^2^ = 0.0093 (1) Å^2^; *R*-factor = 0.001. [A comparison of experimental and fit spectra is shown in Fig. 5 ▸(*b*).] Thus, XAFS reproduced the crystallographic results (*R*_ZnO_ = 1.975 Å, 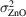 = 0.009 Å^2^) within (pre-determined) intrinsic uncertainties (Δ*R* = ±0.035 Å, Δσ^2^ = ±0.002 Å^2^).

Since the crystallographic results were obtained at *T* = 77 K (Agrawal *et al.*, 2019 ▸), this implies that the coordination chemistry of Zn in M1dr is robust and varies negligibly from *T* = 77 K to 300 K. The role of thermal disorder is concluded to be minimal. This observation unravels a novel perspective of M1dr. M1dr is a Zn metallopeptidase of the M1 Merops family. It is unique in the sense that it is the only two-domain protein amongst the three- and four-domain M1 family peptidases characterized so far (Agrawal *et al.*, 2019 ▸). The reported high-resolution XRD structure for this protein corresponded to non-physiological conditions: 0.2–0.25 *M* ammonium formate, 0.1 *M* bis-tris and 20–27% polyethyl­ene glycol 3350 at pH 5.5 (Agrawal *et al.*, 2019 ▸). In contrast, XAFS of M1dr was measured for pH 7.0, *i.e.* under physiological conditions. The similarity of bond lengths and coordination between XRD and XAFS essentially represents invariance of the Zn coordination chemistry between the two pH conditions. This characteristic is identical to the three- and four-domain proteins of the M1 family. (Zn coordination remains invariant for proteins of different compositions in the M1 peptidase family.) We can therefore conclude a resemblance of M1dr with the three- and four-domain proteins. Strong coordination chemistry may be responsible for the (observed) efficient substrate-binding for M1dr in the absence of the C-domain.

### 3.3. Scope of improvement

XANES and first-shell EXAFS analysis of an ultra-dilute MP solution at beamline BL-9 has been tested to be reliable and feasible in this work. First-shell EXAFS provides information on the metal–ligand unit within a radius *R* ≃ 2 Å (*e.g.* ligand identity, molecular composition and configuration), with implications for disease-marking, binding properties, protein aggregation, multi-site heterogeneity, mutation and cellular catalysis (Smolentsev *et al.*, 2005 ▸; Longa *et al.*, 1999 ▸; Vlasenko *et al.*, 1999 ▸; Sagi *et al.*, 1999 ▸; Katsikini *et al.*, 2009 ▸; Meyer-Klaucke *et al.*, 1999*b* ▸; Bertoncini *et al.*, 1999 ▸). However, interesting science exists beyond the metal–ligand unit, *i.e.* at higher shells (HS) (*R* > 2 Å). For example, electron-spin transport for regulation of chemical reactions and switching behavior is determined by the inter-unit coupling geometry (Giachini *et al.*, 2007 ▸; Kleifield *et al.*, 2001 ▸; Murphy *et al.*, 1997 ▸; Tierney & Schenk, 2014 ▸). Future XAFS experiments of MPs at BL-9 will be designed for the accommodation of such advanced problems. Since HS structural information is contained in the high-frequency component of χ(*k*), it is ultra-sensitive to noise. Success of HS analysis of MPs would therefore mandate high signal statistics. Since our diagnostic tests demonstrated a significant improvement of the signal between single- and four-element SDDs, we conclude that the incident photon flux is sufficient and the statistical problem is related to detection inefficiency. Therefore, employment of a highly efficient multi-element germanium detector at BL-9 can be expected to generate the required statistics for HS analysis. We plan to incorporate micro-focusing and a multi-element germanium detector in the next phase of beamline upgradation.

## 4. Conclusion

We have successfully measured Zn *K*-edge XAFS of analogous synthetic Zn (0.1 m*M*) M1dr solution under *in vivo* conditions at bending-magnet-based beamline BL-9 of Indus-2. Despite a one order-of-magnitude lower metal concentration and the unavailability of a multi-element germanium detector (used in standard XAFS experiments of MPs), we obtained a sufficiently fair spectral quality for reliable first-shell analysis, with strategies such as large sample area, four-element SDD and fast electronics. XAFS results reproduced the Zn^+2^(O/N)_4_ coordination chemistry of the M1dr crystal at *T* = 77 K. This confirmed the feasibility of XAFS of ultra-dilute metalloprotein solutions at BL-9 with the present facilities. Deployment of a standard multi-element Ge detector in the future would significantly enhance the capabilities of this beamline and extend the scope of such work.
